# The yin-yang shaped image following head injury

**DOI:** 10.11604/pamj.2013.16.133.3555

**Published:** 2013-12-09

**Authors:** Ali Akhaddar

**Affiliations:** 1 Department of Neurosurgery, Avicenne Military Hospital, 40000, Marrakech, Morocco; 2 University of Mohammed V-Souissi, 10100, Rabat, Morocco

**Keywords:** Head injury, craniofacial trauma, haematoma, yin-yang

## Image in medicine

A 33-year-old previously healthy man was admitted with a craniofacial trauma sustained in a road traffic accident. No post-traumatic seizures were documented. On physical examination, large wound was observed in the frontal region on the right side. He was comatose, and his consciousness level was 6 on the Glasgow Coma Scale. The right pupil was dilated but responsive to light. The axial slices of the cerebral computed tomography-scan (CT-scan) revealed a distorted right cerebral hemisphere with effacement of the basal cisterns, which was compressed by an extraaxial haematoma located in the right frontoparietal region. This haematoma was found to have two different components: the first (anterior) was epidural and the second (posterior) was subdural. Both haematomas were evacuated through a right frontoparietal craniotomy. Postoperatively, CT-scan showed no evidence of residual haematoma. The patient has been in good health throughout the 3-month follow-up since the accident. Classic CT-scan appearance of intracranial epidural haematoma (EDH) is high density biconvex shape adjacent to the skull and usually confined to small segment of calvaria. On the contrary, acute subdural haematoma (ASDH) appear more diffuse, less uniform, usually concave over brain surface and often less dense (from mixing with cerebrospinal fluid) than EDH. But when combined, these two different surgical entities are not easy to distinguish on CT-scan. The Yin-yang-shaped image seen in our patient is formed by an anterior biconvex shape and a posterior concave shape, the combination of adjacent epidural and acute subdural haematoma. The radiologic features of our presentation are interesting and might be useful to distinguish both adjacent EDH and ASDH.

**Figure 1:**
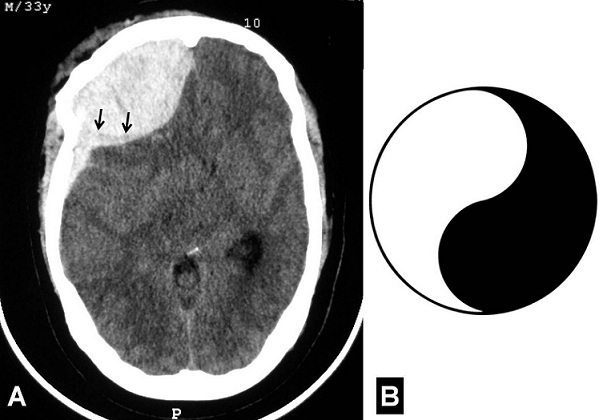
A) Axial CT-scan showing extraaxial hemorrhagic lesion on the right frontoparietal region. This lesion has two different components: the first (anterior) was epidural and the second (posterior) was subdural. Note the limit within this lesion (arrows); B) The Yin-yang symbol or Taijitu

